# Supplementary material for: A Comprehensive Sequencing Analysis of Testis-Born miRNAs in Immature and Mature Indigenous Wandong Cattle (Bos taurus)
Source: Genes (Basel). 2022 Nov 23;13(12):2185. doi: 10.3390/genes13122185 (PMC9777600; doi:10.3390/genes13122185)
Supplement: Supplementary file 1 [file genes-13-02185-s001.zip › genes-1999853-supplementary.pdf]

Figure S1. The workflow represents the small RNA library construction for the Illumina.

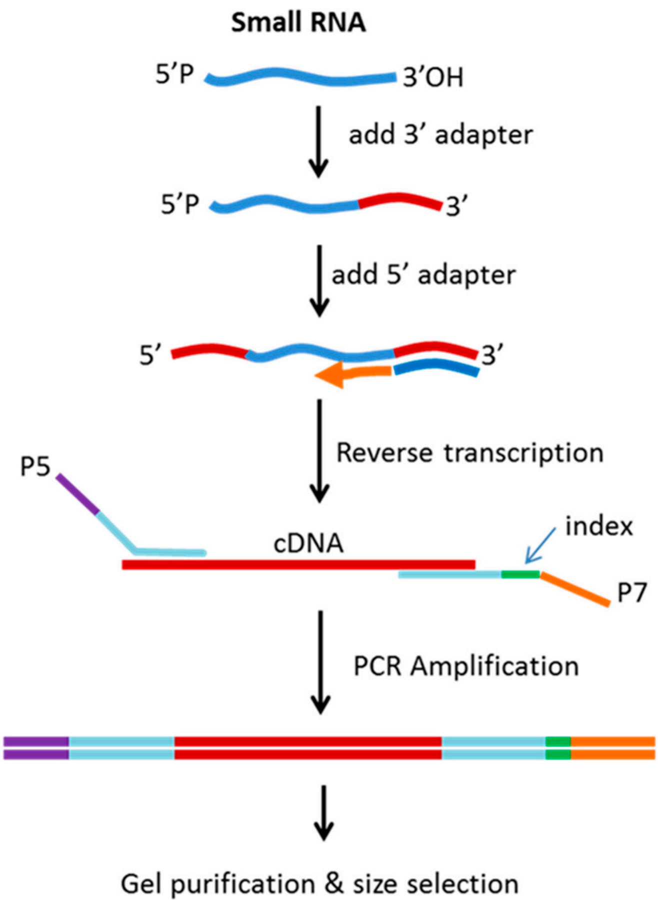

Figure S2. Schematic represented the mi-RNAs analysis and adopted route.

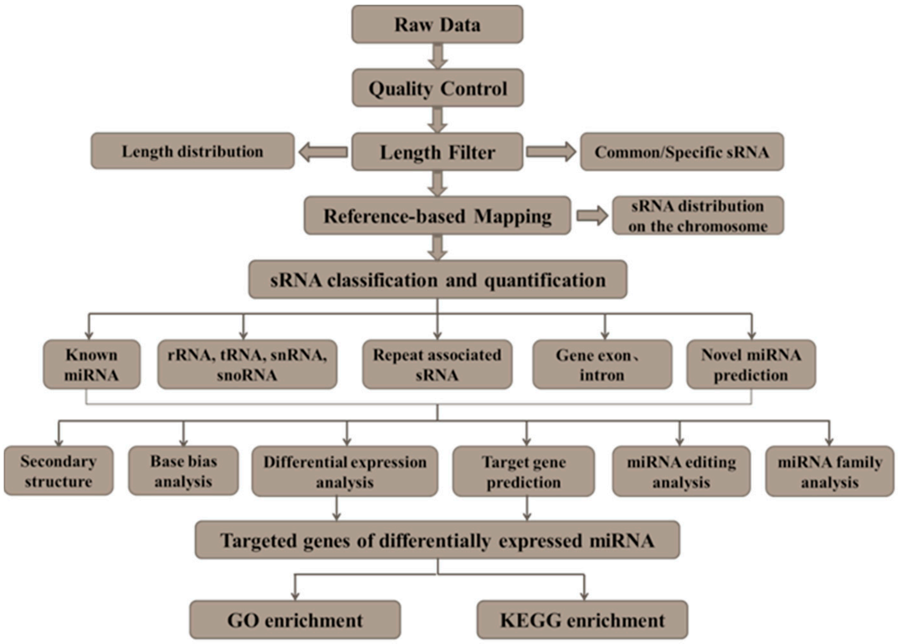

Table S1. The total raw reads and classifications of undesirable read.

Sample	Total-reads	Containing-N	Low- quality	5-adapter contamination	3-adapter-null	Nucleotide bases	Clean reads
Bull_2	19243007 (100.00%)	0 (0.00%)	35234 (0.18%)	2793 (0.01%)	53151 (0.28%)	89576 (0.47%)	19062253 (99.06%)
Calf_1	17862525 (100.00%)	0 (0.00%)	80873 (0.45%)	3702 (0.02%)	160809 (0.90%)	15067 (0.08%)	17602074 (98.54%)
Bull_1	23004713 (100.00%)	0 (0.00%)	98942 (0.43%)	3273 (0.01%)	153339 (0.67%)	110432 (0.48%)	22638727 (98.41%)
Calf_3	21796590 (100.00%)	0 (0.00%)	132023 (0.61%)	3717 (0.02%)	223213 (1.02%)	36314 (0.17%)	21401323 (98.19%)
Calf_2	17696661 (100.00%)	0 (0.00%)	28895 (0.16%)	3592 (0.02%)	101313 (0.57%)	20338 (0.11%)	17542523 (99.13%)
Bull_3	21312453 (100.00%)	0 (0.00%)	133560 (0.63%)	4074 (0.02%)	213242 (1.00%)	145994 (0.69%)	20815583 (97.67%)
